# Clinical Diagnostic Utility of IP-10 and LAM Antigen Levels for the Diagnosis of Tuberculous Pleural Effusions in a High Burden Setting

**DOI:** 10.1371/journal.pone.0004689

**Published:** 2009-03-11

**Authors:** Keertan Dheda, Richard N. Van-Zyl Smit, Leonardo A. Sechi, Motasim Badri, Richard Meldau, Gregory Symons, Hoosein Khalfey, Igshaan Carr, Alice Maredza, Rodney Dawson, Helen Wainright, Andrew Whitelaw, Eric D. Bateman, Alimuddin Zumla

**Affiliations:** 1 Lung Infection and Immunity Unit & CTBRI, UCT Lung Institute & Division of Pulmonology, Department of Medicine, University of Cape Town, Cape Town, South Africa; 2 Institute of Infectious Diseases and Molecular Medicine, University of Cape Town, Cape Town, South Africa; 3 Centre for Infectious Diseases and International Health, University College Medical School, London, United Kingdom; 4 Department of Biomedical Sciences, University of Sassari, Sardinia, Italy; 5 National Health Laboratory Service, Cape Town, South Africa; 6 Division of Medical Microbiology, Department of Clinical Laboratory Sciences, University of Cape Town, Cape Town, South Africa; National Institute for Infectious Diseases (INMI) L. Spallanzani, Italy

## Abstract

**Background:**

Current tools for the diagnosis of tuberculosis pleural effusions are sub-optimal. Data about the value of new diagnostic technologies are limited, particularly, in high burden settings. Preliminary case control studies have identified IFN-γ-inducible-10kDa protein (IP-10) as a promising diagnostic marker; however, its diagnostic utility in a day-to-day clinical setting is unclear. Detection of LAM antigen has not previously been evaluated in pleural fluid.

**Methods:**

We investigated the comparative diagnostic utility of established (adenosine deaminase [ADA]), more recent (standardized nucleic-acid-amplification-test [NAAT]) and newer technologies (a standardized LAM mycobacterial antigen-detection assay and IP-10 levels) for the evaluation of pleural effusions in 78 consecutively recruited South African tuberculosis suspects. All consenting participants underwent pleural biopsy unless contra-indicated or refused. The reference standard comprised culture positivity for *M. tuberculosis* or histology suggestive of tuberculosis.

**Principal Findings:**

Of 74 evaluable subjects 48, 7 and 19 had definite, probable and non-TB, respectively. IP-10 levels were significantly higher in TB vs non-TB participants (p<0.0001). The respective outcomes [sensitivity, specificity, PPV, NPV %] for the different diagnostic modalities were: ADA at the 30 IU/L cut-point [96; 69; 90; 85], NAAT [6; 93; 67; 28], IP-10 at the 28,170 pg/ml ROC-derived cut-point [80; 82; 91; 64], and IP-10 at the 4035 pg/ml cut-point [100; 53; 83; 100]. Thus IP-10, using the ROC-derived cut-point, missed ∼20% of TB cases and mis-diagnosed ∼20% of non-TB cases. By contrast, when a lower cut-point was used a negative test excluded TB. The NAAT had a poor sensitivity but high specificity. LAM antigen-detection was not diagnostically useful.

**Conclusion:**

Although IP-10, like ADA, has sub-optimal specificity, it may be a clinically useful rule-out test for tuberculous pleural effusions. Larger multi-centric studies are now required to confirm our findings.

## Introduction

Annually, over half a million pleural effusions are diagnosed world-wide and it is one of the commonest forms of extra-pulmonary tuberculosis (TB; [1]). In Africa, where TB is out of control, extrapulmonary TB is more common due to HIV co-infection. The diagnosis of TB pleural effusion is challenging. Pleural biopsy has a good yield (∼80%) but it is invasive, expensive and requires trained medical personnel [2]. Smear microscopy of pleural fluid had a dismal yield (∼5%) and culture takes several weeks to obtain [2]. Other rapid diagnostic tools such as nucleic acid amplification tests (NAAT) have poor sensitivity in pleural fluid (∼50%; [3]), though the performance outcomes of a standardized NAAT has not previously been evaluated in a high HIV sero-prevalence setting [3]. Given the drawbacks of existing tools investigators have pursued the detection of measurable biomarkers, including IFN-γ levels [4] and adenosine-deaminase (ADA), as diagnostic adjuncts [2]. However, measuring IFN-γ is relatively expensive in high burden settings [5] and ADA is not widely available in clinical laboratories, and is non-specific even in high burden settings [2], [6].

An alternative promising, but poorly studied, biomarker is IFN-γ inducible protein of 10 kDa (IP-10). IP-10, a Th1-associated chemokine, was found to be a useful discriminatory tool in three case-controlled studies [7], [8], [9] but its clinical diagnostic utility in an unselected cohort of TB suspects is unknown. More recently, lipoarabinomannan (LAM) antigen detection was found to be useful for the diagnosis of TB when using urine obtained from Tanzanian TB suspects [10]. However, the utility of a standardized LAM antigen-capture ELISA assay (Clearview® TB *ELISA*) has not previously been evaluated in other body compartments including pleural fluid.

In this study we prospectively evaluated the comparative diagnostic utility of established (adenosine deaminase [ADA]), more recent (standardized nucleic-acid-amplification-test [NAAT]) and newer technologies (IP-10 levels and a standardized LAM mycobacterial antigen-detection assay) for the evaluation of pleural effusions in 78 South African tuberculosis suspects. The gold standard for tuberculosis was culture positivity for *M. tuberculosis* and/ or histology in keeping with tuberculosis.

## Methods

### Patient recruitment, characterization and routine laboratory tests

**Figure 1:**
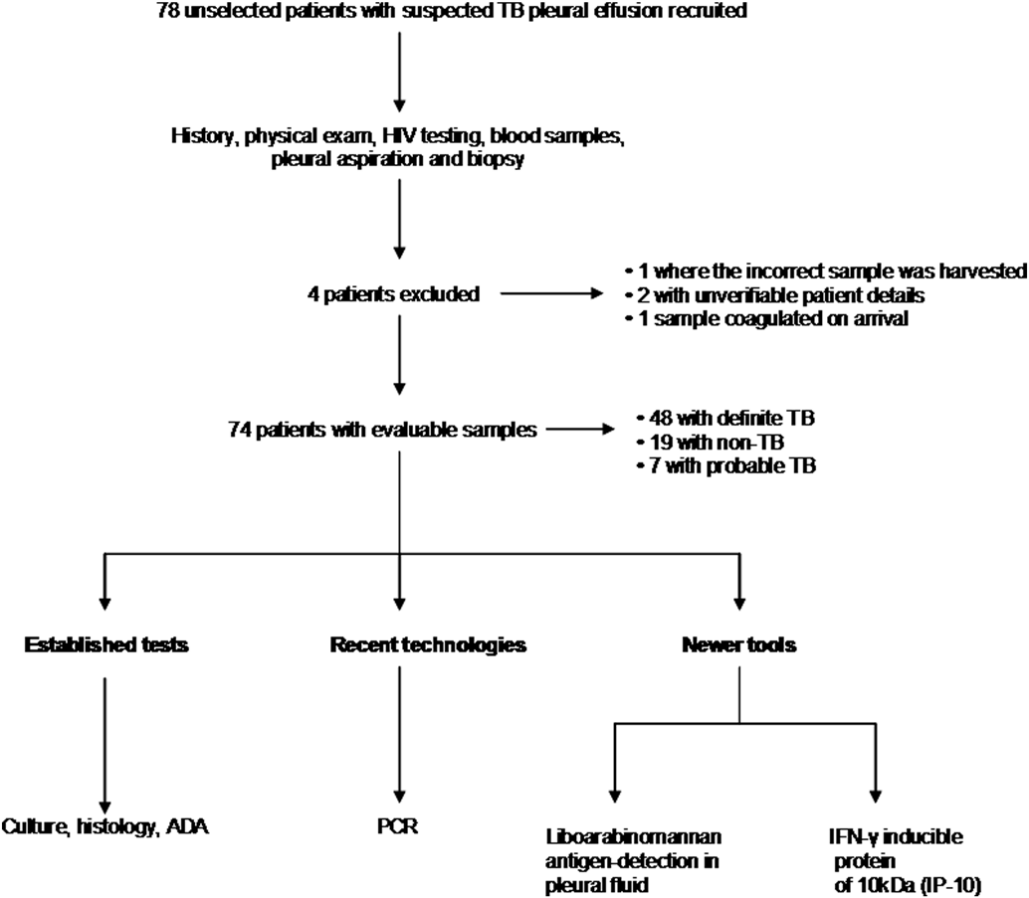
Summary and flow chart of the established and newer technologies evaluated.

Seventy-eight consecutive patients with suspected TB pleural effusion (persistent fever, night sweats or cough, chest pain, loss of weight, previous tuberculosis or recent TB contact, or any patient in whom, due to suggestive symptoms or signs, TB was part of the differential diagnosis) were prospectively recruited at the Groote Schuur, Somerset and Victoria hospitals in Cape Town, South Africa, after informed consent (see figure 1 for an overview of the study plan), over a 12 month period (ending 30 April 2008). Those under 18 years of age, pregnant or refusing consent were not recruited. The data presented here is part of a parent study, which evaluated the role of unstimulated IFN-γ versus quantitative T cell responses for the diagnosis of TB pleural effusion. Four patients were excluded for other reasons and thus there were 74 patients with evaluable results (figure 1). Study approval was obtained from the University of Cape Town Health Sciences Faculty research ethics committee.

All patients had a history taken, detailed physical examination performed, routine haemtological investigations, including testing for HIV infection, chest x-ray, sputum examination when possible (fluorescent microscopy for acid fast bacilli and mycobacterial culture using the MGIT 960 system), and aspiration of approximately 20 ml of pleural fluid (or closest obtainable volume) for biochemical (protein and glucose), cytological (for malignant cells, cell differential [Dif-Quick, American Scientific products]), and microbiological (Gram stain and culture for bacterial pathogens, mycobacterial fluorescence microscopy and culture for *M. tuberculosis* using the MGIT 960 system) evaluation.

For accurate characterization of disease multiple closed pleural biopsies (approximately four) were undertaken using an Abraham's needle under local anesthesia, by a trained internal medicine resident (specialist registrar). In 16 patients biopsies were not performed because of patient refusal, a contra-indication or a positive culture of fluid, or histology from another site, prior to attempted pleural biopsy. The reference standard for tuberculosis was culture positivity for *M. tuberculosis* and/ or histology in keeping with tuberculosis (caseous necrosis or acid fast bacilli with or without granuloma formation). Patients were thus characterized as (i) definite TB: either positive *M.tb* culture (sputum, pleural fluid or tissue) and/ or histology in keeping with tuberculosis, and a clinico-radiological picture consistent with TB with clinical response to anti-TB treatment; (ii) non-TB: alternative diagnosis made on histology or pleural fluid aspiration, and not treated for TB, and on 3 to 6 month follow-up there were no features to suggest TB, and (iii) probable TB: clinical picture of TB but not satisfying the definite TB criteria and treated for TB by the attending physician.

### ADA, LAM antigen-detection, NAAT and IP-10 levels

Pleural fluid protein and ADA levels were derived using the Biuret and colorimetric methods, respectively. LAM antigen concentration in the pleural fluid was measured in duplicate, after a heating step to dissociate antigen and antibody, according to the manufacturer's instructions (Clearview® TB *ELISA*, ME, USA; see http://www.clearview.com/tb_elisa.aspx). An interim analysis was done in the 1^st^ 24 recruited patients (14 definite or probable TB cases and 10 non-TB cases) to use a go/ no-go decision point for further LAM testing. For nucleic-acid-amplification the Amplified Tuberculosis Direct Test (Genprobe, San Diego, CA) was performed in duplicate according to the manufacturer's instructions (H37Rv served as a positive control and *M. intracellulare* served as the negative control) and readouts were obtained using a Leader 50 Luminometer (Genprobe, San Diego, CA).

IP-10 levels were measured, according to the manufacturer's instructions, using a standardized Human IP-10 ELISA Kit (Hycult Biotechnology, Uden, The Netherlands). All assays were performed by an experienced laboratory technician who was blinded to patient and clinical details.

### Bio-clinical score

To ascertain the relative value of newer tests in a high burden setting, regression models were fitted to identify variables independently associated with risk of tuberculosis, taking into account findings from the history, physical examination and pleural fluid biochemical data. The final bio-clinical scoring rule, incorporating age and protein levels, was developed by assigning a relative score or points to each of the variables included in the final multivariate model. Here we use the model to evaluate the relative incremental value of the different pleural diagnostic tests.

### Statistical analysis

Categorical variables were compared using the χ^2^ test or Fisher exact test and continuous variables were compared using t-student test, whenever appropriate. Non-parametric tests (Mann-Whitney) were used for non-normally distributed variables. Concordance between tests was measured using the kappa co-efficient. Diagnostic accuracy, including 95% confidence intervals, was assessed using sensitivity, specificity, predictive values and area under the ROC in the TB and non-TB sub-groups. The study report was prepared using the Standards for Reporting of Diagnostic Accuracy (STARD initiative) format (19).

## Results

### Demographic, clinical and biochemical data

A summary of the study plan is shown in figure 1. Of the 78 patients recruited 4 were excluded from the analysis (one because of a coagulated sample, one because an incorrect sample had been harvested [ascitic instead of pleural fluid], and two due to unverifiable patient details). Thus 74 patients had results for pleural fluid microscopy, biochemistry, ADA, PCR or IP-10 levels. There were 48, 19 and 7 patients with definite, non-TB and probable TB, respectively. Effusions in the non-TB group were due to several causes (2 lymphoma, 2 myeloproliferative disorders, 9 adeno or small cell carcinoma, 3 parapneumonic, and 3 due to other causes).

Of those tested 20/51 (49%) were HIV positive. In the TB vs non-TB group the mean age (years) was significantly lower (37 vs 55, respectively; p<0.0001) though there were more people of Black African and mixed race (48 vs 19, respectively; p = 0.03). There was no significant inter-group difference for sex, HIV status, BCG vaccination or employment status. The mean (SD) pleural fluid protein levels were significantly higher in the TB vs non-TB group [58.9(15.7) vs 43.4(18.5), respectively; p = 0.003]. In the final multivariate logistic regression model, age (<42 years), [odds ratio (OR) = 3.89, 95% CI 1.01–14.90, p = 0.04] and protein levels (>53 g/L) [OR = 3.59, 95% CI 1.02–12.56, p = 0.04) were independently associated with the risk of tuberculosis. These variables, when incorporated into a bio-clinical score, had a maximal sensitivity and specificity of 54 and 89%. The median (25^th^; 75^th^ percentiles) pleural fluid cell count was 1.75×10^6^ cells/ml (1.03; 5.45×10^6^ cells/ml) and the median volume of fluid obtained was 20 ml (10; 25 ml).

### Results of smear, culture, histology and NAAT

**Figure 2:**
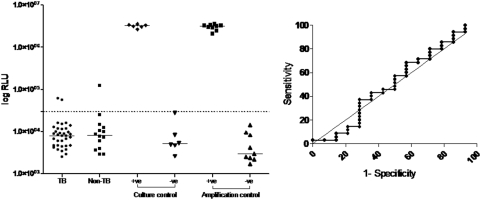
Scatter-plots (left panel) and area under the ROC (right panel) of a standardized nucleic-acid amplification test using pleural fluid from patients with tuberculous (TB) and non-tuberculous (non-TB) effusions. Area under the ROC values are shown in table 1. Positive (culture and amplification) and negative controls (culture and amplification) from 6 independent runs are shown.

Smear, pleural fluid culture, and biopsy (tissue culture and histology) were positive in 1, 27 and 41 of the 48 definite TB cases, respectively, and by definition, in none of the non-TB cases. None of the probable TB cases were culture or biopsy positive but all were treated empirically for TB based on clinical suspicion. Twenty one percent (16/ 74) of patients did not have a pleural biopsy (refused by 4 patients, contra-indicated in 2 patients, 1 in whom a liver biopsy was done, and 9 in whom the culture result was positive prior to a biopsy being done [6 sputum culture positive and 3 pleural fluid culture positive]). The diagnostic outcomes of protein levels at different cut-points is shown in table 1. Performance outcomes of the NAAT (manufacturer-derived cut-point) were poor and are shown in table 1, table 2 and figure 2.

**Table 1:** Performance outcomes of ADA, microbiological investigations, a nucleic-acid-amplification-test [NAAT] and unstimulated IFN-γ levels for the diagnosis of TB pleural effusion in 74 TB suspects using the *definite and non-TB* groups.

	Cut-point	Sens %	Spec %	PPV %	NPV %	Accuracy	AUC
Maximum clinical score	n/a	54 (40; 67)	89 (68;97)	92 (77; 98)	43 (29;59)	64 (52;74)	n/a
Protein	>30 g/l**	94 (83;98)	12 (3; 34)	75 (62; 84)	40 (12; 77)	72 (60; 81)	0.72
	>60 g/l***	46 (33; 60)	94 (78; 99)	94 (79; 99)	39 (26; 54)	59 (47; 70)	
ADA	>30 iu/l**	96 (86; 99)	69 (44; 86)	90 (78; 96)	85 (58; 96)	89 (78; 94)	0.93
	>47 iu/l***	91 (80; 98)	94 (72; 99)	98 (85; 99)	79 (57; 92)	92 (82; 97)	
	>13 iu/l#	100 (92;100)	38 (19;62)	82 (70;90)	100 (61;100)	84 (73;91)	
NAAT	30 000 RLU	6 (2;18)	93 (69.99)	67 (9;91)	28 (17;42)	29 (18;42)	0.50
IP-10	28170 pg/ml***	80 (64; 91)	82 (57; 97)	91 (78;97)	64 (43;80)	81 (69;89)	0.82
IP-10	4035 pg/ml#	100 (91;100)	53 (31;74)	83 (70;91)	100 (71;100)	86 (75;93)	0.77

( ; ) = 95% CI.

** Cut-point used in day-to-day clinical practice in Cape Town, South Africa, where the test guides the institution of anti-TB treatment.

AUC = area under the ROC curve.

*** AUC-derived cut-point.

# cut-point with a high NPV.

RLU = relative light units detectable using a luminometer.

**Table 2:** Performance outcomes of ADA, microbiological investigations, a nucleic-acid-amplification-test [NAAT] and unstimulated IFN-γ levels for the diagnosis of TB pleural effusion in 74 TB suspects when the *definite and probable TB groups are combined*.

	Cut-point	Sens %	Spec %	PPV %	NPV %	Accuracy	AUC
Maximum clinical score	n/a	52	94	97	38	62	67
		39;65	73;99	83;99	25;53	50;72	
Protein	>30 g/l**	94	18	79	50	76	0.72
		(85;98)	(6;41)	(67;87)	19;81	(65;85)	
	>60 g/l***	44	94	79	35	56	
		(32;58)	(73;99)	(81;99)	(23;49)	45;67	
ADA	>30 iu/l**	94	65	89	79	87	0.94
		85;98	41;83	79;95	52;92	77;93	
	>47 iu/l***	89	65	80	71	78	
		77;95	41;83	68;88	50;86	67;85	
	>13 iu/l#	100	35	83	100	84	
		93;100	17;59	52;90	61;100	74;91	
NAAT	30 000 RLU	7	93	75	26	29	0.49
		3; 19	69;99	30;95	16;39	19;42	
IP-10	28170 pg/ml#	79	83	92	61	80	0.82
		(63;89)	(59;94)	(79;97)	(41;78)	(68;88)	
IP-10		100	53	83	100	86	0.77
	4035 pg/ml#	(91;100)	(31;74)	(70;91)	(71;100)	(75;93)	

( ; ) = 95% CI.

** Cut-point used in day-to-day clinical practice in Cape Town, South Africa, where the test guides the institution of anti-TB treatment.

AUC = area under the ROC curve.

*** AUC-derived cut-point.

# cut-point with a high NPV.

RLU = relative light units detectable using a luminometer.

### IP-10 and ADA

**Figure 3:**
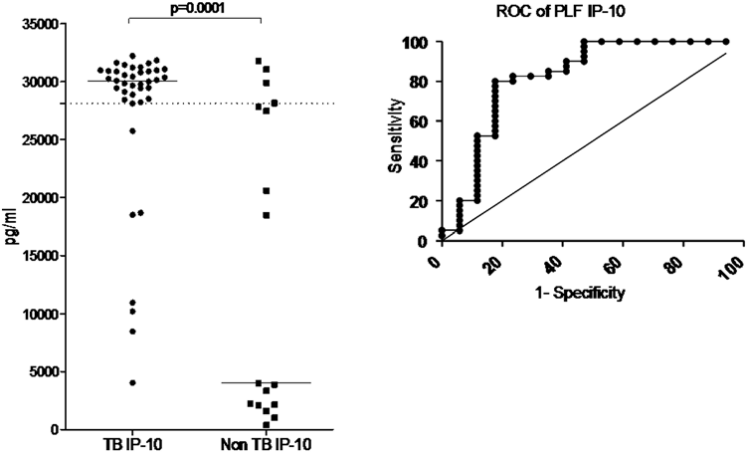
Scatter-plot (left panel) showing IP-10 levels in patients with pleural tuberculosis versus non-tuberculosis controls. The area under the ROC curve was 0.82.

The IP-10 scatter-plot (n = 73) and area under the ROC is shown in figure 3 and performance outcomes are shown in table 1. At an AUC-derived cut-point of 28170 pg/ml (definite vs non-TB) the sensitivity (%), specificity, +LR (likelihood ratio) and −ve LR (95% CI) was 80 (64; 91), 82 (57; 97), 4.53 (2.32; 8.85) and 0.24 (0.19; 0.32), respectively, and the area under the ROC curve was 0.82. By contrast, at a lower cut-point of 4035 pg/ ml (derived from the ROC for the best NPV) the sensitivity was 100 (91; 100), specificity 53 (31; 74), NPV (100; 71; 100), +LR 2.13 (1.66; 2.72) and −ve LR 0 (0; 0), respectively. Also shown are the outcomes when the probable TB cases were included in the analysis. HIV status did not impact on IP-10 or NAAT test results (data not shown).

By contrast the ADA (30 iu/l cut-point) had a sensitivity of 96 (86; 99), specificity 69 (44; 86), NPV 85 (58; 96), +LR 17.4 (2.44; 124.1), and −ve LR 0.09 (0.05; 0.15), respectively. At the cut-point used for clinical decision making in Cape Town (30 iu/l) ADA had a sub-optimal specificity of 64% compared to a higher AUC-derived cut-point (47 iu/l; table 1, table 2).

### LAM antigen

We measured LAM antigen levels (Clearview® TB *ELISA*, ME, USA) in the first 22 TB suspects recruited (12 TB, 2 probable TB and 10 non-TB). The sensitivity of LAM antigen was 8% (1 of 12 patients) and specificity was 100%.

### Incremental diagnostic value of different tests

Incremental test value is summarised in table 3. ADA and IP-10 (at both cut-points) was more sensitive than the clinical evaluation alone, though histology did not add any further value. Adding IP-10 to ADA had little incremental benefit, however, at the lower IP-10 cut-point the % NPV (95% CI) improved from 79 (57; 92) for ADA alone, and 82 (59; 94) for ADA +IP-10, to 100 (71; 100).

**Table 3:** Individual and incremental value of different test combinations.

Combination	Sens	Spec	PPV	NPV	accuracy
Clinical score$	54 (40; 67)	89 (68; 97)	92 (77; 98)	43 (29; 59)	64 (52; 74)
Clinical score+histology	91 (78; 96)	63 (42; 78)	81 (86; 89)	79 (57; 92)	81 (69; 88)
Clinical score+ADA	96 (85; 99)	81 (60; 92)	92 (81; 97)	89 (89; 97)	91 (82; 96)
Clinical score+histology+ADA	92 (81; 97)	83 (61; 94)	94 (83; 98)	79 (57; 92)	89 (79; 95)
ADA*	91 (80; 98)	94 (72; 99)	98 (85; 99)	79 (57; 92)	92 (82; 97)
IP-10	80 (64; 91)	82 (57; 97)	91 (78;97)	64 (43;80)	81 (69;89)
IP-10 (4035 pg/ml cut-point)	100 (91;100)	53 (31;74)	83 (70;91)	100 (71;100)	86 (75;93)
IP-10 and clinical score	38 (25;52)	95 (73;99)	94 (74;99)	39 (26;53)	55 (49;66)
IP-10+ADA	93 (81; 98)	88 (64; 97)	95 (84; 99)	82 (59; 94)	91 (81; 96)

* = 47 iu/l cut-point and non-asterisked values refer to the 30 iu/l cut-point.

$ = maximal clinical score. Unless, otherwise stated all IP-10 outcomes refer to the 28170 pg/ml cut-point.

## Discussion

The diagnostic yield of current tools for pleural effusion is sub-optimal. In this study we evaluated IP-10 and LAM antigen detection against other tools for the diagnosis of TB pleural effusion. Although IP-10 levels were significantly higher in TB compared to non-TB patients it was not clinically useful because of the sub-optimal specificity in the non-TB group. In three case control studies, two immunological [8], [9] and one diagnostic [7], IP-10 levels were significantly higher in the TB compared to the control group. In the only study reporting diagnostic outcomes in 11 TB patients [7], IP-10 discriminated well between TB and malignant PE, and performed as well as unstimulated IFN-γ (AUC of 0.93 vs 0.99, respectively). Comparatively, although we found highly significant inter-group differences (TB vs non-TB; p<0.0001;), IP-10 missed the diagnosis in ∼20% of TB patients and was falsely positive in ∼20% of non-TB patients using the AUC-derived cut-point. Thus, IP-10 was not a meaningful clinical discriminatory tool and levels were highly variable in patients with malignant and para-pneumonic effusions.

IP-10 is an IFN-γ-driven chemokine and a non-specific Th1 inflammatory marker and thus can be elevated in other disorders including pulmonary fibrosis [11], multiple sclerosis [12] and lymphoma [13]. It is also well recognized that case control studies, though useful for preliminary screening evaluation of new biomarkers, tends to overestimate diagnostic performance outcomes [14]. Moreover, statistical inter-group difference, as we demonstrate, does not necessary imply clinical usefulness. By contrast, at the lower cut-point the IP-10 NPV was 100%, and the negative LR was 0.24, making it a promising rule out test. This may be useful in clinical practice, particularly in undiagnosed pleural effusions when the histology is non-specific or non-representative of pleural tissue. In this context a negative IP-10 test may prompt a more vigorous search for other pathologies (e.g. using alternative diagnostic strategies including thoracoscopy), and reduce exposure to unwarranted or empiric anti-TB treatment and its attendant toxicity.

By comparison the ADA (30 iu/l cut-point) had a better sensitivity, PPV and positive LR, though the specificity and NPV was sub-optimal implying that ∼3 in 10 non-TB subjects would be erroneously treated for TB and ∼1 in 7 patients with a negative test would in fact have TB. At a higher cut-point (47 iu/l) the former misdiagnosis would be reduced, but not eliminated, and at the expense of missing ∼1 in 10 TB cases. At the lower cut-point ADA would be an excellent rule-out test but specificity would be poor. Nevertheless, ADA is cheaper and more widely available than IP-10. Thus IP-10, which can be measured with several commercially available ELISA assays, cannot replace ADA. Rather, it could useful in a specific clinical context where ruling out TB would be useful.

In the parent study using the same cohort of patients we show that unstimulated IFN-γ levels very accurately distinguishes TB from non-TB effusions in African patients (data not shown). This preliminary analysis showed a modest correlation between IP-10 and IFN-γ levels (Spearman r = 0.3836). Why does IP-10, an IFN-γ inducible chemokine, not correlate highly with IFN-γ levels, as it does in peripheral blood [15]? IP-10 is also regulated by other cytokines including IFN-α, IL-1β and IL-12 [16], [17], [18], disease phenotype can modulate cytokine half-life [19], and there may be differential cellular uptake and cellular regulation of IP-10 [17]. To meaningfully evaluate the relative clinical value IP-10 and ADA we compared their utility to a simple bio-clinical score, generated through regression analysis, and relevant to a resource-poor setting [20], [21]. Both IP-10 and ADA substantially improved the sensitivity and NPV compared to bio-clinical assessment alone. A combination of the clinical score with ADA improved the NPV but not to an extent that was clinically useful. A combination of the clinical score with IP-10, or IP-10 and ADA, added little incremental value. At the lower IP-10 cut-point NPV was still better than ADA at either cut-point (low or high). We also investigated the effect of HIV status on test performance outcomes. IP-10 levels were not significantly different in HIV+ and negative patients, though HIV+ patients were more likely to have a positive pleural fluid culture (non-significant; data not shown).

TB antigen detection has previously been investigated for its diagnostic utility in pleural effusions. However, tuberculostearic acid was found to have limited diagnostic utility [22]. More recently, a preliminary study from Africa suggested that detection of urinary LAM antigen was a useful diagnostic adjunct in TB suspects [10]. The assay has now been commercialized into a finalized prototype (see http://www.clearview.com/tb_elisa.aspx), which we evaluated in pleural fluid. For cost-containment purposes further testing of LAM was discontinued, after an interim analysis of the first 24 patient results, because of its poor diagnostic utility (only one out of 14 definite or probable TB cases tested positive for LAM antigen). Why LAM antigen virtually undetectable in pleural fluid? Preliminary experiments excluded technical reasons and batch variability. To exclude the lack of antigen-protein dissociation (pleural fluid has a high protein content that may bind free LAM) a heating step was incorporated into the test protocol to ensure dissociation. The lack of LAM antigen detection in the majority of TB pleural effusions probably reflects the pauci-bacillary nature of pleural disease, though high affinity antigen-antibody binding cannot entirely be excluded.

The variability of NAAT performance outcomes are highly dependant on laboratory protocols and we therefore evaluated a standardized NAAT, hitherto not undertaken in an African setting, in pleural TB suspects. The sensitivity of commercial NAATs for pleural TB are highly variable (20 to 100%; summarized in detail in [3], [23]). Although, in our study, specificity was high the sensitivity was poor (only 6%). Putative reasons may include the paucibaciliary nature of the disease, inhibitors in the pleural fluid, and sub-optimal mycobacterial nucleic-acid extraction efficiency. The latter is less likely as a culture control (∼10 organisms) was included in the experiment. However, the assay used has no inhibitor-specific internal positive control. Further studies are required to assess the impact of HIV-infection and inhibitors on TB-specific nucleic acid amplification tests in high burden settings.

We took several steps to minimize bias and ensure study validity, including consecutive recruitment with universally applied and pre-specified inclusion criteria, an experienced technician blinded to clinical details, invasive procedures to ensure accurate classification of patient and control sub-groups, and use of a pre-specified reference standard. We also provide, through comparison with clinical assessment and incremental test value over existing tools, information on clinical utility rather than test performance outcomes only (sensitivity, specificity etc; [24]). Thus, there is an emphasis on ‘diagnostic’ rather than ‘test’ research. The sensitivity of the test is compared to the sensitivity of clinical assessment, which enables the incremental value of the test over the clinician's assessment, to be determined. Therefore, a test with an apparently high sensitivity may have limited clinical utility if it has little or no incremental benefit over clinical assessment. It is also meaningful, for the purposes of determining value, to determine the incremental benefit of a new test over an existing one. Nevertheless, this study has several limitations. The sample size was small and accuracy estimates relatively imprecise. We also evaluated the test in the same population used to determine the cut-points. Further work is now required to validate these cut-points in different populations. Also, results are generalisable only to high burden settings; further and larger studies are required to evaluate whether outcomes are different in low burden settings. For example, the high background rate of LTBI, host biological factors and early TB infection may have impacted on test results.

In conclusion, like ADA, IP-10 levels at the AUC-derived cut-point are not specific for tuberculosis, though at the lower cut-point they appear to be promising rule-out tests for TB in a high burden setting. Larger studies are required in other settings to confirm these findings.
